# Serotonin Inhibition of Claustrum Projection Neurons: Ionic Mechanism, Receptor Subtypes and Consequences for Claustrum Computation

**DOI:** 10.3390/cells13231980

**Published:** 2024-11-29

**Authors:** Kelly Li Lin Wong, Martin Graf, George J. Augustine

**Affiliations:** 1 Neuroscience & Mental Health Program, Lee Kong Chian School of Medicine, Nanyang Technological University, Singapore 308232, Singapore; kellywlilin@gmail.com (K.L.L.W.); martin_graf@tll.org.sg (M.G.); 2 Temasek Life Sciences Laboratory, Singapore 117604, Singapore

**Keywords:** serotonin, claustrum, neuromodulation

## Abstract

The claustrum is a small but densely interconnected brain structure that is innervated by axons containing serotonin (5-HT), a neuromodulator that has been implicated in control of sleep and in the actions of psychedelic drugs. However, little is known about how 5-HT influences the claustrum. We have combined whole-cell patch-clamp measurements of ionic currents, flash photolysis, and receptor pharmacology to characterize the 5-HT responses of individual claustral projection neurons (PNs) in mouse brain slices. Serotonin application elicited a long-lasting outward current in claustral PNs. This current was due to an increase in membrane permeability to K^+^ ions and was mediated mainly by the type 1A 5-HT receptor (5-HTR-1A). The 5-HT-induced K^+^ current hyperpolarized, and thereby inhibited, the PNs by reducing action potential firing. Focal uncaging of 5-HT revealed that inhibitory 5-HTR-1As were located at both the soma and dendrites of PNs. We conclude that 5-HT creates a net inhibition in the claustrum, an action that should decrease claustrum sensitivity to excitatory input from other brain areas and thereby contribute to 5-HT action in the brain.

## 1. Introduction

The claustrum is a long and irregular sheet of neurons nestled between the insula and striatum. Because the claustrum is heavily interconnected with many other brain regions in organisms ranging from mice to humans [1,2,3,4,5,6], the claustrum has been likened to a cortical conductor [7]. While the function of the claustrum is beginning to come into focus [8,9,10,11], much less is known about how claustrum activity is regulated by neuromodulators [12].

One potential modulator of claustrum function is serotonin (5-HT). The release of 5-HT within the brain is closely tied to sleep–wake states: 5-HT is higher in wake states than sleep states [13,14,15]. Emerging evidence indicates the involvement of the claustrum in sleep; claustral activity is higher in sleep states than wake states [16,17,18]. This anticorrelation between claustral activity and 5-HT release suggests that 5-HT inhibits the claustrum during wake states, while a lack of 5-HT should enable claustral activity during sleep states.

Such serotonergic modulation of the claustrum is strongly suggested by 5-HT receptor (5-HTR) expression [19,20,21,22,23,24,25,26,27,28] and input from major serotonergic nuclei [4,17,29,30,31,32,33]. More recently, the claustrum was shown to be involved in the actions of the psychedelic drugs 2,5-Dimethoxy-4-iodoamphetamine (DOI; [34,35]) and psilocybin [36], which are thought to act via 5-HTRs [37]. Further, the claustrum has been implicated in the loss of consciousness linked to lesions of the dorsal raphe nuclei, a major source of serotonergic input throughout the brain [38].

Here, we investigated how 5-HT influences claustral projection neurons (PNs), the principal output cells of the claustrum. Claustral PN responses to 5-HT were detected using whole-cell patch-clamp recordings of PN activity in mouse brain slices. By locally applying 5-HT onto claustral PNs, we found that 5-HT inhibits these neurons. Further experiments identified the ionic mechanisms and 5-HTRs mediating these serotonergic inhibitory responses. Finally, we photolyzed caged 5-HT to determine the location of the 5-HTRs responsible for serotonergic inhibition. Together, these results provide a detailed characterization of the inhibitory actions of 5-HT on claustral output neurons.

## 2. Materials and Methods

### 2.1. Animals

Approximately 100 wild-type mice (C57BL/6 strain) of both sexes and approximately 2 months of age were used for the experiments. All animal procedures used were approved by the Institutional Animal Care and Use Committees of NTU and TLL.

### 2.2. Brain Slice Preparation

As described in Chia et al. [39] and Graf et al. [38], mice were sacrificed by deep anesthesia (isoflurane) and were subsequently decapitated. Their brains were swiftly isolated and transferred to an ice-cold cutting solution containing (in mM) 250 sucrose, 26 NaHCO_3_, 10 D-glucose, 4 MgCl_2_, 3 myo-inositol, 2.5 KCl, 2 sodium pyruvate, 1.25 NaH_2_PO_4_, 0.5 ascorbic acid, 0.1 CaCl_2_, and 1 kynurenic acid (350–360 mOsm, pH 7.4). Using a vibratome (VT1200S, Leica, Wetzlar, Germany), coronal acute brain slices of 250 µm thickness were obtained over anterior–posterior coordinates of approximately +1.7 mm (before Bregma) to −0.6 mm. The brain slices were incubated at 34 °C for 30 min in an artificial cerebral spinal fluid (ACSF) external solution containing (in mM) 126 NaCl, 24 NaHCO_3_, 1 NaH_2_PO_4_, 2.5 KCl, 2 CaCl_2_, 2 MgCl_2_, 10 glucose, and 0.4 ascorbic acid (300–310 mOsm, pH 7.4). The slices were kept at room temperature (24 °C) in ACSF post-incubation. All solutions were bubbled with carbogen (95% oxygen and 5% carbon dioxide). All chemicals were purchased from Sigma Aldrich except for NaH_2_PO_4_ (Kanto Chemicals, Tokyo, Japan).

### 2.3. Electrophysiological Recordings

Borosilicate glass pipettes (1B150F-4, WPI, Sarasota, FL, USA) were prepared using a pipette puller (PC-10, Narashige, Tokyo, Japan). Whole-cell patch-clamp recordings were performed on claustral neurons at room temperature using glass pipettes (3–8 MΩ resistance) filled with a K-gluconate internal solution containing (in mM) 130 mM K-gluconate,10 KOH, 2.5 MgCl_2_, 10 HEPES, 4 Na_2_ATP, 0.4 Na_3_GTP, 5 EGTA-disodium, 5 Na_2_ phosphocreatine, and 0.2% neurobiotin (290–295 mOsm, pH 7.4). All reagents were purchased from Sigma Aldrich except for KOH (Kanto Chemical) and neurobiotin (#SP-1120, Vector Laboratories, Newark, CA, USA). The slices were perfused with carbogen-oxygenated ACSF at a rate of 1 mL/min. Neurons were visualized by differential interference imaging on a two-photon microscope (FV1200-MPE, Olympus, Tokyo, Japan). Recordings were obtained from individual PNs, using a MultiClamp 700B amplifier (Molecular Devices, San Jose, CA, USA), and digitized with a DigiData 1440 interface (Molecular Devices) sampling at 50 kHz. All membrane potentials stated in this paper have been corrected for a liquid–liquid junction potential of 11.7 mV. Criteria for the inclusion of patch-clamp measurements were as follows: an access resistance (R_a_) less than 30 MΩ, a ratio of R_a_ to membrane resistance less than 20%, and/or a resting membrane potential more negative than −50 mV. Electrical recordings were primarily analyzed using Clampfit 10.7 (Molecular Devices). Voltage-clamp measurements of 5-HT-induced membrane currents were low-pass filtered (8-pole Bessel Filter, 20 Hz).

### 2.4. Classification of Claustral Neurons

Neurons were depolarized with a series of rectangular current pulses (1 s duration) to evoke action potentials. The resulting voltage traces were then analyzed to determine numerous resting and active electrical properties, with extracted parameters then entered into a trained classifier that used these intrinsic electrical properties to identify the neurons as one of eight distinct neuron types [40]. These neurons included five types of projection neurons (PN1 to PN5) and three interneuron types (PV, SST, and VIP); the current paper focusses on the much more abundant projection neurons. These recordings were performed in the absence of synaptic blocking drugs to emulate the conditions used by Graf et al. [40].

### 2.5. Responses to 5-HT Application

To examine the 5-HT responses of individual claustral neurons, a solution of 5-HT (100 µM) dissolved in 0.1% DMSO and ACSF was locally applied via a glass pipette (2–4 MΩ) whose tip was positioned ~50 µm away from the neuron. Positive pressure (100 ms, 1 bar) was applied via a Picospritzer II (Parker Hannifin, Cleveland, OH, USA). In most experiments, neurons were voltage-clamped and held at holding potential of −70 mV, with kynurenic acid (100 µM) and GABAzine (10 µM; Tocris, Singapore) added to the external solution to eliminate the possible polysynaptic effects of 5-HT. Because not all neurons responded to 5-HT, applications were repeated at least 3 times to determine whether or not a 5-HT response was present, with an interval of 60 s between applications to ensure sufficient time for 5-HT responses to recover between applications. To determine the reversal potential of the 5-HT response of each neuron, the neuron was held at membrane potentials ranging from −110 to −70 mV. 5-HTR antagonists were added to the external solution at the following concentrations: 5-HTR-1A (WAY-100635; 1 µM), 5-HTR-2A (MDL-11939; 5 µM), and 5-HTR-2C (RS-102221; 5 µM). Measurements were usually made at least 10 min after applying drug-containing solutions to the brain slices. All drugs were purchased from Sigma Aldrich (Singapore) unless stated otherwise. Several of these drugs were relatively hydrophobic; these were dissolved in DMSO prior to dilution with ACSF. In all cases, the final DSMO concentration was no more than 0.1% *v*/*v*.

In some current-clamp experiments, neurons were depolarized using two identical current ramps (5 s duration, 400 pA peak amplitude) spaced 30 s apart. To avoid possible run-down of AP firing over prolonged recording times, responses to the first current ramp served as controls for responses evoked by the second current ramp. 5-HT was then applied at a specific time before the start of the second current ramp, typically 5 s unless otherwise indicated. The same paradigm was used when rectangular current pulses (1 s duration) were used instead of ramps.

### 2.6. 5-HT Uncaging

To examine the spatial distribution of 5-HTRs on claustrum neurons, caged 5-HT was focally photolyzed. Several versions of caged 5-HT have been synthesized, including RuBi-5-HT [41] and NPEC-5-HT [42,43]. We used BHQ-O-5-HT because of its optimal spectral separation from the fluorescent dye that was employed to visualize neuronal structure and stability [44,45]. In these experiments, the external solution was oxygenated ACSF containing the caged 5-HT compound BHQ-O-5-HT (10 µM; Kerafast, Boston, MA, USA), which was recirculated using a peristaltic pump system. Caged 5-HT in the solution was photolyzed using a 405 nm laser (~32 mW). The area over which 5-HT was uncaged was determined by visualizing the structure of each neuron. For this purpose, Alexa 594 dye (50 µM; Invitrogen, Carlsbad, CA) was added to the internal solution and a 2-photon z-stack image of the dye-filled neuron was obtained. 5-HT was then uncaged over various compartments of a neuron: (1) whole-neuron responses were generated by a 100 µm-by-100 µm photolysis area that was centered over the soma; (2) somatic responses were evoked by a smaller 10 µm-by-10 µm area over the soma; and (3) dendritic responses were evoked by a 100 µm-by-100 µm area placed over the dendrites, 100 µm away from the soma.

Responses to uncaged 5-HT were measured by voltage-clamping neurons at a holding potential of −30 mV to optimize the amplitude of the small 5-HT-induced outward currents (the reversal potential of these currents was −95 mV). Responses from 3 to 5 trials were averaged to improve response signal-to-noise ratio.

### 2.7. Immunohistochemistry

**Figure 1:**
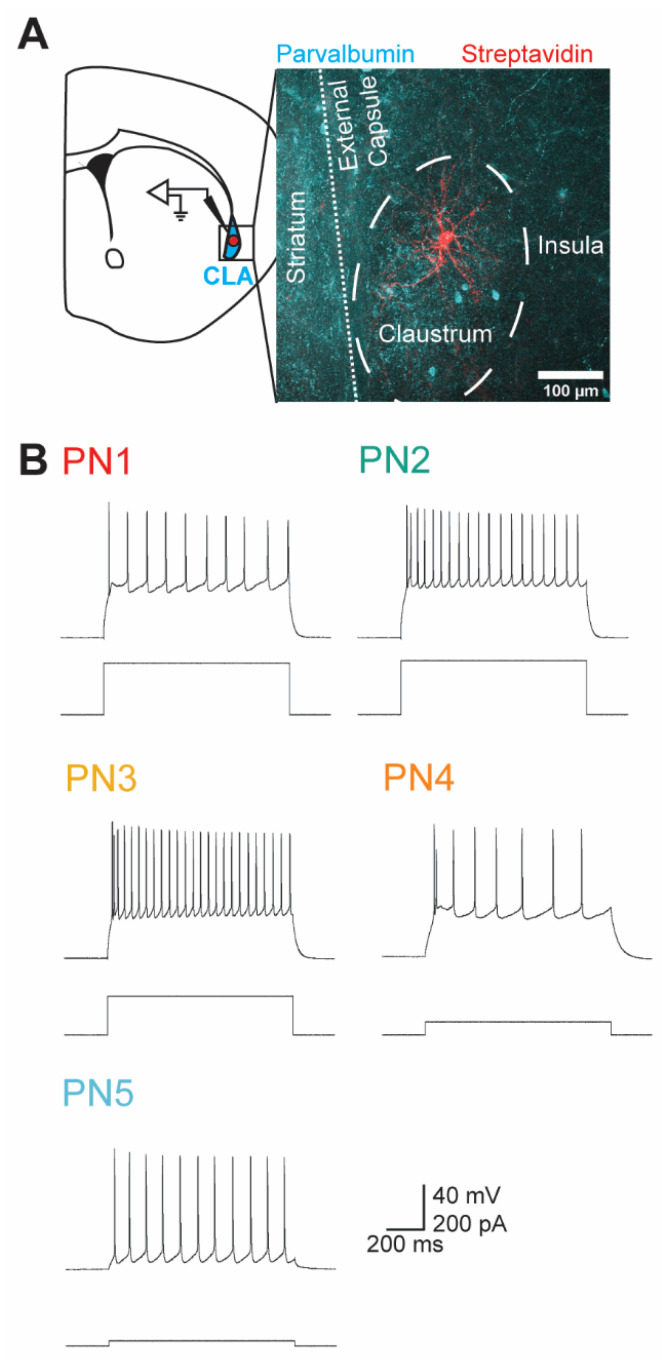
Identifying claustral projection neurons via their intrinsic electrical properties. (**A**) A whole-cell patch-clamp recording from a single claustral neuron in a coronal brain slice. The neuron was filled with neurobiotin, which was labeled by streptavidin (red). The claustrum (CLA) core is identifiable as a PV-rich (cyan) elliptical structure located between the insula and the striatum. The striatum also highly expresses PV but is separated from the cortex by the external capsule. (**B**) Representative recordings of AP firing (upper traces) evoked by depolarizing currents (lower traces) in PN subtypes PN1 to PN5.

Following electrophysiological recordings, brain slices were processed for parvabumin (PV) immunohistochemistry to identify the location of patched neurons (Figure 1). If the soma of a patched neuron was within or <50 µm beyond the PV-enriched claustrum core, it was considered to reside within the claustrum [40]. The slices were fixed with 4% paraformaldehyde for 2 h and then washed thrice (20 min each time) with phosphate-buffered saline (PBS; BUF-2041-10X, Axil Scientific, Singapore) containing 0.25% Triton X-100 (PBST; #H5141, Promega, Madison, WI, USA). Slices were subsequently blocked with 5% goat serum (#G9023, Sigma Aldrich) in PBST and incubated with the following antibodies in PBST with 1% goat serum overnight at 4 °C: 1:800 rabbit anti-PV (#PV-235, Swant, Burgdorf, Switzerland) and 1:1000 streptavidin conjugated with Alexa-633 (#S21375, Invitrogen). The slices were again with washed with PBST thrice (20 min each) and incubated with a 1:1000 goat anti-rabbit antibody conjugated with Alexa-488 (#A28175, Invitrogen) for 2 h. Slices were washed with PBS and mounted on glass slides with Dako mounting media (#CS70330-2, Agilent Technologies, Santa Clara, CA, USA). All histological processing steps were performed at room temperature on an orbital shaker, unless otherwise indicated.

### 2.8. Image Acquisition

All fluorescence images were obtained using either a two-photon microscope (FV1200-MPE, Olympus) or a widefield microscope (Axioscan Z1, Zeiss, Oberkochen, Germany). Images were then processed and/or analyzed primarily on FIJI [46]. Z-stack projections were obtained using either standard deviation or maximum intensity projection methods.

### 2.9. Calculations and Analyses

All statistical analyses were performed using Origin (Origin Lab; Northampton, MA, USA) or R [47] and are reported in Supplementary Table S1. Levene’s test for homogeneity was performed before deciding on the appropriate statistical test to compare means. All values shown represent the mean and standard error of the mean (SEM) for the indicated parameters.

As will be shown in the Results section, 5-HT-induced currents were determined to be carried by potassium (K^+^) ions because their reversal potential (the membrane potential at which the currents reverse polarity) was similar to the equilibrium potential of potassium ($E_{K}$) [48]. $E_{K}$ was calculated from the Nernst equation:

(1) $$V_{Eq}=\frac{RT}{zF}ln\left(\frac{{\left[K^{+}\right]}_{out}}{{\left[K^{+}\right]}_{in}}\right)$$

where R is the universal gas constant, T is the temperature in degrees Kelvin, z is the ionic valence (+1 for K^+^), F is Faraday’s constant, and [K^+^]_in/out_ are the K^+^ concentrations inside and outside the neuron. For an ohmic K^+^ current, the underlying chord conductance can be calculated as follows:

(2) $$g=I/(V_{h}-E_{K}),$$

where I is the peak amplitude of the 5-HT-induced current and $V_{h}$ is the holding potential.

When analyzing the results of current-clamp experiments, the current threshold was the current input that elicited the first action potential (AP), while half-width was the duration at the half-maximal amplitude of the first AP. The input–output relationship of a neuron was defined as the relationship between AP frequency and amount of depolarizing current applied. It was calculated for each 250 ms epoch during a 5 s depolarizing current ramp. A Boltzmann function was fitted to input–output curves:

(3) $$y=\frac{A_{1}-A_{2}}{1+e^{(x-x_{0})/dx}}+A_{2}$$

where $A_{1}$ is the minimum AP frequency (when no depolarizing current is applied), $A_{2}$ is maximal AP frequency, the half-maximal AP frequency is $(A_{1}+A_{2})/2$, $X_{0}$ is the amount of current needed to evoke the half-maximal AP frequency, and the slope of the function is $(A_{2}-A_{1})/4dx$.

The relationship between the magnitude of the 5-HT response and the distance from the soma was used to determine the spatial resolution of 5-HT uncaging. When uncaging 5-HT near the soma of a neuron, the distance between the center of 10 µm-by-10 µm uncaging areas and the center of the soma was measured. When uncaging occurred over 100 µm-by-100 µm areas, the distance between the center of the uncaging area and the closest process of the neuron was measured. Using the ImageJ framework for quantifying neuronal structure (SNT), via the FIJI plugin subscription *NeuroAnatomy* [49], neuronal dendrites were traced to create a binary mask. The distance from center of the uncaging area to the nearest process of the neuron was then determined from a Sholl analysis of the binary mask.

## 3. Results

### 3.1. Identification of Claustral PNs

We performed whole-cell patch-clamp recordings of the electrical signals of claustral neurons in mouse brain slices. Because the claustrum is a small structure that is challenging to visualize in unstained brain slices, post hoc immunohistochemistry was used to confirm that all patched neurons were within the parvalbumin-rich core of the claustrum. For this purpose, neurons were filled with neurobiotin via the patch pipette; this allowed the neurons to be identified via streptavidin staining, while the claustrum was identified by anti-parvalbumin antibody staining (Figure 1A).

Like all other brain regions, the claustrum contains a heterogeneous mixture of neurons [40]. Previous work from our lab has established that there are eight subtypes of claustral neurons that can be split into two major groups—projection neurons (PNs) and interneurons—based on their intrinsic electrical properties [40]. We employed the classification scheme of Graf et al. [40] to distinguish claustral PNs from interneurons and further categorized the PNs into five subtypes based on a variety of electrical properties. These properties included the amplitude of depolarizing afterpotentials, the threshold current for evoking an action potential (AP), and the temporal pattern of AP firing in response to prolonged depolarizations, among other parameters. Examples of APs evoked by depolarization of each type of PN are shown in Figure 1B.

### 3.2. Claustral PNs Are Inhibited by a K^+^ Conductance Increase

To assess the actions of 5-HT on claustral PNs, 5-HT was applied onto patched, identified PNs. Serotonin was applied via pressure ejection, and possible polysynaptic responses were eliminated by treatment with blockers of glutamate receptors (kynurenic acid; KA) and GABA receptors (GABAzine; GBZ). A concentration of 100 µM serotonin, which is ten times the saturating dose reported in most published dose–response curves [50,51,52], was applied to elicit maximal responses. To measure ionic currents induced by 5-HT application, neurons were voltage-clamped at a holding potential of −70 mV.

**Figure 2:**
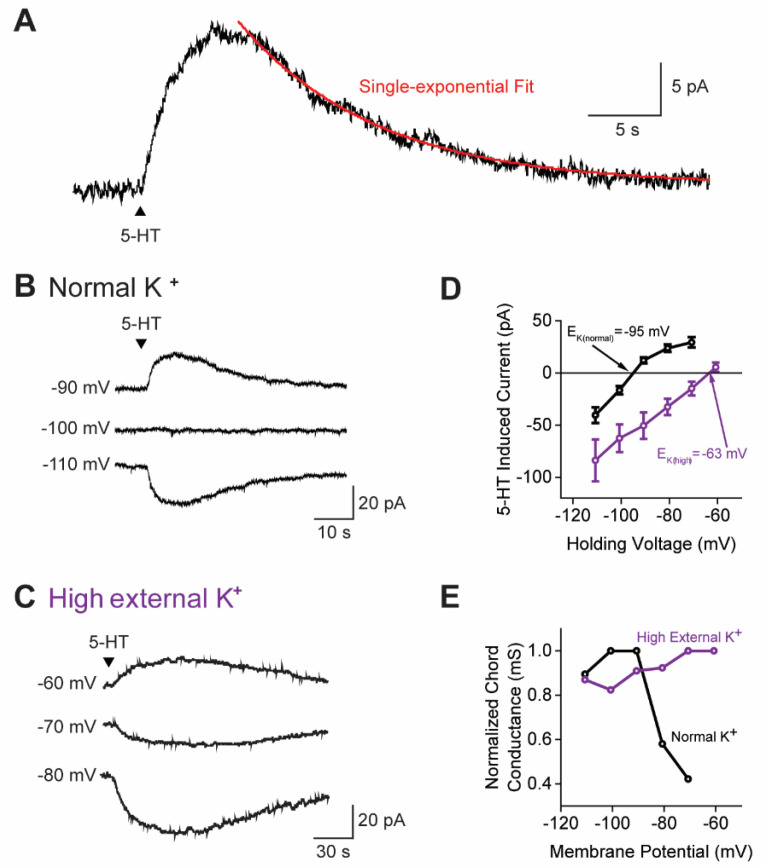
A K^+^ conductance increase mediated the 5-HT response of claustrum PNs. (**A**) Representative current induced in a claustrum PN in response to local application of 5-HT (100 µM; at arrowhead). The time course of current decay was fitted with a single-exponential function (red). (**B**) Currents induced by 5-HT in a claustral PN held at different membrane potentials. In this cell, the 5-HT-induced current reversed its polarity at −100 mV. (**C**) Currents induced by 5-HT in a claustral PN held at various membrane potentials and bathed in a high external K^+^ solution. In this cell, the 5-HT-induced current reversed its polarity between −60 and −70 mV. (**D**) Relationships between membrane potential and 5-HT-induced currents in normal (black; *n* = 10) and high external K^+^ (purple *n* = 6) solutions. The 5-HT responses were sensitive to the electrochemical gradient of K^+^ and reversed at −95 mV and −63 mV, respectively. Points indicate mean values, and error bars show SEM. (**E**) Normalized chord conductances, calculated from Equation (2), of 5-HT responses measured in normal (black) and high external K^+^ solution (purple). Points indicate mean values.

Under these conditions, 5-HT induced a slow and long-lasting outward current in 67.2% (123/182) of claustral PNs (Figure 2A). On average, the outward current peaked at 6.3 ± 0.3 s (mean ± SEM) after the onset of 5-HT application and reached a maximum amplitude of 22.1 ± 1.7 pA. This outward current decayed gradually, following a single-exponential time course (red line in Figure 2A), with a mean time constant of 19.3 ± 2.2 s. The total charge associated with the 5-HT response was 284 ± 35 pC. Notably, even in the absence of KA and GABAzine, an outward current of similar magnitude was observed (*n* = 13), suggesting that the 5-HT-induced outward current had either a minimal or no poly-synaptic component (Supplementary Figure S1).

Long-lasting, 5-HT-induced currents are often mediated by K^+^ channels [53,54,55,56]. To determine whether K^+^ mediated the 5-HT-induced outward current that we observed, an ion-substitution experiment was conducted. Altering the external concentration of K^+^ around claustral PNs changed the electrochemical gradient and equilibrium potential of K^+^ (E_K_), consequently shifting the reversal potential (E_rev_) of the 5-HT-induced currents. With a normal extracellular K^+^ concentration, the 5-HT-induced current reversed at −95 mV (Figure 2B; *n* = 10). When the external K^+^ concentration was increased four-fold, the 5-HT-induced current reversed at −63 mV (Figure 2C; *n* = 6). In both conditions, the E_rev_ closely matched the calculated E_K_ of −103 mV and −67 mV, respectively (Figure 2D) [40]. Furthermore, since the slope of the current–voltage curves was positive, the 5-HT-induced current could be attributed to an increase in K^+^ conductance. The chord conductance of the 5-HT response was calculated from Equation (2). In normal extracellular K^+^ conditions, the chord conductance decreased beyond E_rev_, while it was constant at the same potentials when E_K_ was shifted in elevated extracellular K^+^ conditions (Figure 2E). Such properties indicate that 5-HT activates an inwardly rectifying K^+^ conductance [53,54,55]. Because the E_rev_ for the K^+^ current was more negative than the action potential threshold (approximately −35 mV) [37,38], this 5-HT response should inhibit claustral PNs.

### 3.3. Claustral PN Subtypes Differ in Probability of 5-HT Responses

**Figure 3:**
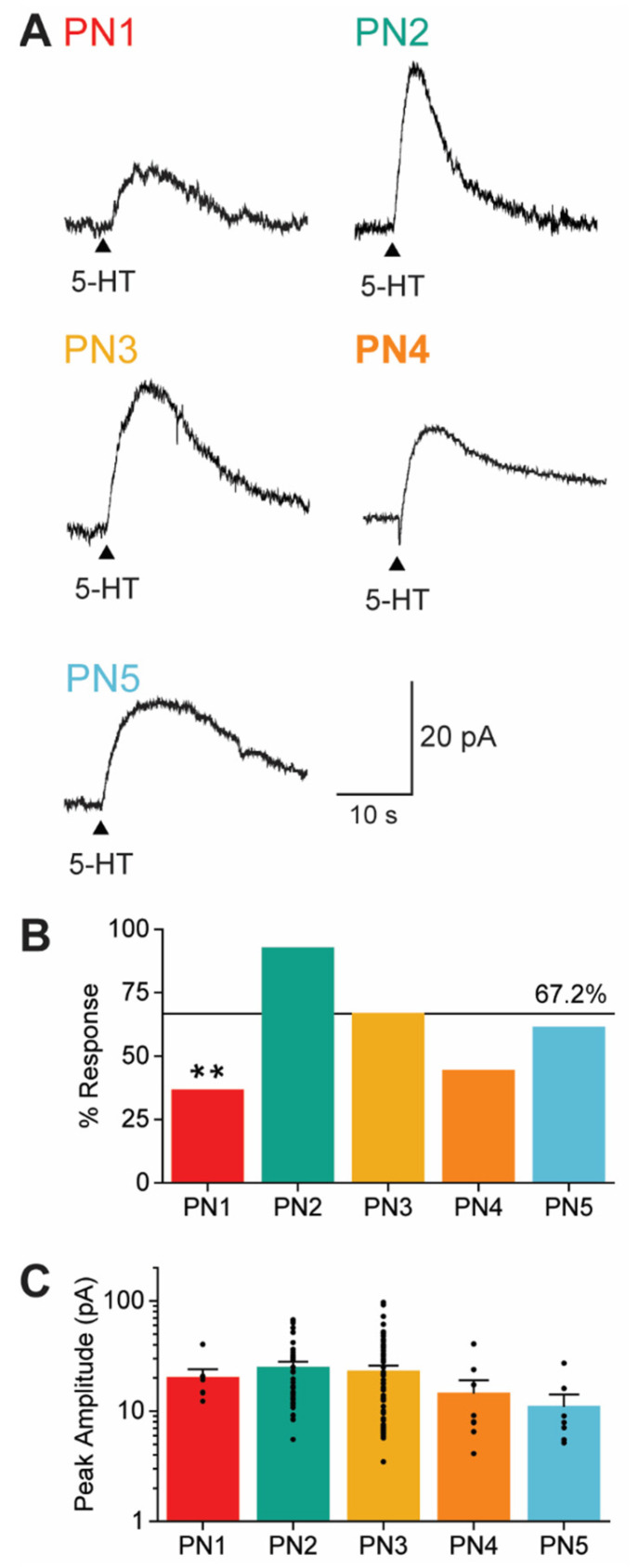
5-HT induced K^+^ currents in all claustral PN subtypes. (**A**) Representative traces of 5-HT-induced outward currents in the five PN subtypes. The black arrowhead indicates the timing of pressure application of 5-HT (100 µM). (**B**) Response rates of the five PN subtypes. The horizontal line represents the mean response rate (67.2%) for all PNs. Asterisks indicate that PN1 cells have a significantly lower rate of responding to 5-HT (*p* = 0.008; see Supplementary Table S1). (**C**) The peak amplitude of the 5-HT-induced outward current was not significantly different across the 5 PN subtypes (refer to Supplementary Table S1 for statistical analysis). Points show individual measurements, bars indicate mean values, and error bars show SEM. ** *p* ≤ 0.01.

We next asked whether the response to 5-HT varied across the five subtypes of PNs. All PN subtypes generated outward currents in response to 5-HT application (Figure 3A). The only statistically significant difference among these subtypes was the probability of evoking a response (Figure 3B); PN2 neurons showed the highest rate of response to 5-HT (92.9%, 39/42), while PN1 had the lowest response rate (36.8%, 7/19). There were no significant pairwise differences between the PN subtypes in terms of the peak amplitude of their 5-HT-induced currents (Figure 3C), nor were there differences in other response parameters such as the magnitude of 5-HT-induced charge, the rate of current decay, or the time to peak response (Supplementary Table S1). Thus, while all claustral PN subtypes exhibited very similar 5-HT-induced responses, the probability of them responding varied between subtypes.

### 3.4. 5-HT Responses Are Generated by Multiple Types of 5-HTRs

5-HT modulates neuronal activity by binding to members of the 5-HTR family [57,58]. The slow time course of the 5-HT-induced K^+^ conductance suggests the involvement of metabotropic 5-HTRs rather than ionotropic 5-HT3 receptors, which typically mediate rapid excitatory responses. We examined the possible roles of several candidate 5-HTR subtypes, specifically the 1A, 2A, and 2C subtypes, which were identified based on their expression in the claustrum and their established roles in 5-HT signaling in other neurons [19,20,21,22,23,24,25,26,27,28,59,60].

**Figure 4:**
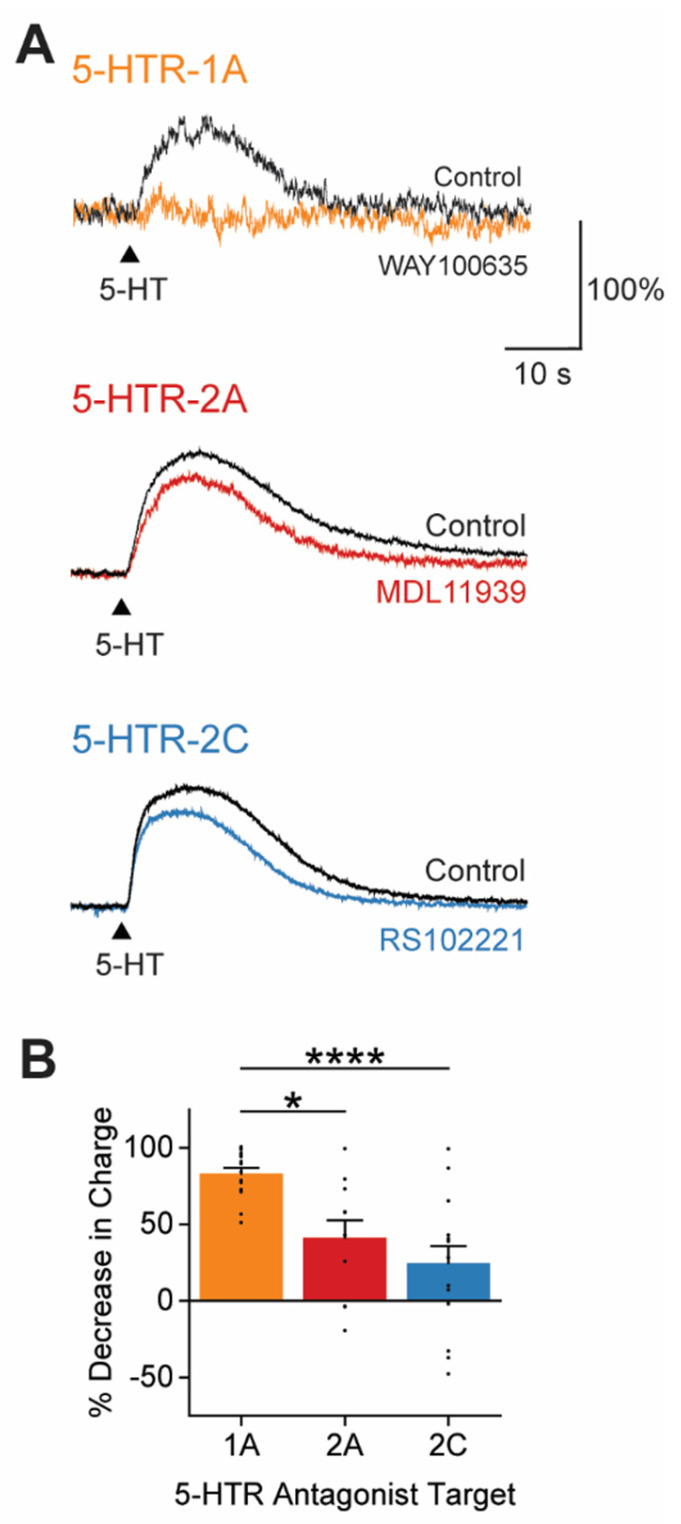
5-HT responses were generated by multiple types of 5-HTRs. (**A**) Representative responses to 5-HT (100 µM) before (Control, black) and after application of the indicated 5-HTR antagonists: 5-HTR-1A antagonist WAY100635 (1 µM, orange), 5-HTR2A antagonist MDL11939 (5 µM, red), and 5-HTR-2C antagonist RS102221 (5 µM, blue). The amplitudes of control responses are normalized to 100% to illustrate the magnitude of the block by each type of antagonist. (**B**) Decrease in the 5-HT-induced response of PNs, measured as response charge, following application of each 5-HTR antagonist. Points show individual measurements, bars indicate mean values, and error bars show SEM. Asterisks indicate statistically significant differences; refer to Supplementary Table S1 for statistical analyses. * *p* ≤ 0.05, **** *p* ≤ 0.0001.

To determine which 5-HTRs mediate the inhibitory action of 5-HT on claustral PNs, we compared the 5-HT-induced K^+^ current of individual neurons before and after applying subtype-specific antagonists (Figure 4A). WAY100635, a selective 5-HTR-1A antagonist [61,62], reduced the mean 5-HT response by 83.1% (*n* = 16). Thus, 5-HTR-1A plays a predominant role in mediating the 5-HT-induced K^+^ current. A 5-HTR-2A antagonist, MDL11939 [63,64], decreased the mean 5-HT response by 41.2% (*n* = 11), indicating the partial involvement of this receptor as well. Further, the 5-HTR-2C antagonist RS102221 [65,66,67] also reduced the mean 5-HT response, in this case by 24.7% (*n* = 15). Thus, ranked according to the ability of antagonists to block the 5-HT-induced K^+^ current (Figure 4B), the claustrum 5-HT response was primarily mediated by 5-HTR subtypes 1A, 2A, and 2C. Although our sample size was too limited to draw firm conclusions, we did not observe any statistically significant differences in the sensitivity of different claustrum PN subtypes to these 5-HTR antagonists (Supplementary Figure S2). This suggests that heterogeneity in the degree of antagonist blockade of 5-HT responses seen in Figure 4B was not a consequence of PN subtype heterogeneity.

It is notable that the combined amount of blockade produced by the 5-HTR-1A, 2A, and 2C antagonists exceeded 100%. This suggests that claustral PNs may express varying compositions of 5-HTRs, with 5-HTR-1A likely being the predominant subtype. Additionally, it could be related to drug crosstalk. Although MDL11939 is at least 100 times more selective for 5-HTR-2A than 5-HTR-2C, and RS102221 shows the reverse selectivity [68], RS102221 exhibits low levels of binding to 5-HTR-2A at the concentration used in these experiments [69]. Similarly, MDL11939, despite having the lowest affinity for 5-HTR-2C among commonly used 5-HTR-2A antagonists [70], may also bind to 5-HTR-2C at the concentration used. Therefore, the 41.2% block produced by MDL11939 likely represents the upper bound of the 5-HT effect mediated by the combined actions of 5-HTR-2A and 5-HTR-2C.

### 3.5. Actions of 5-HT on Action Potential Firing

**Figure 5:**
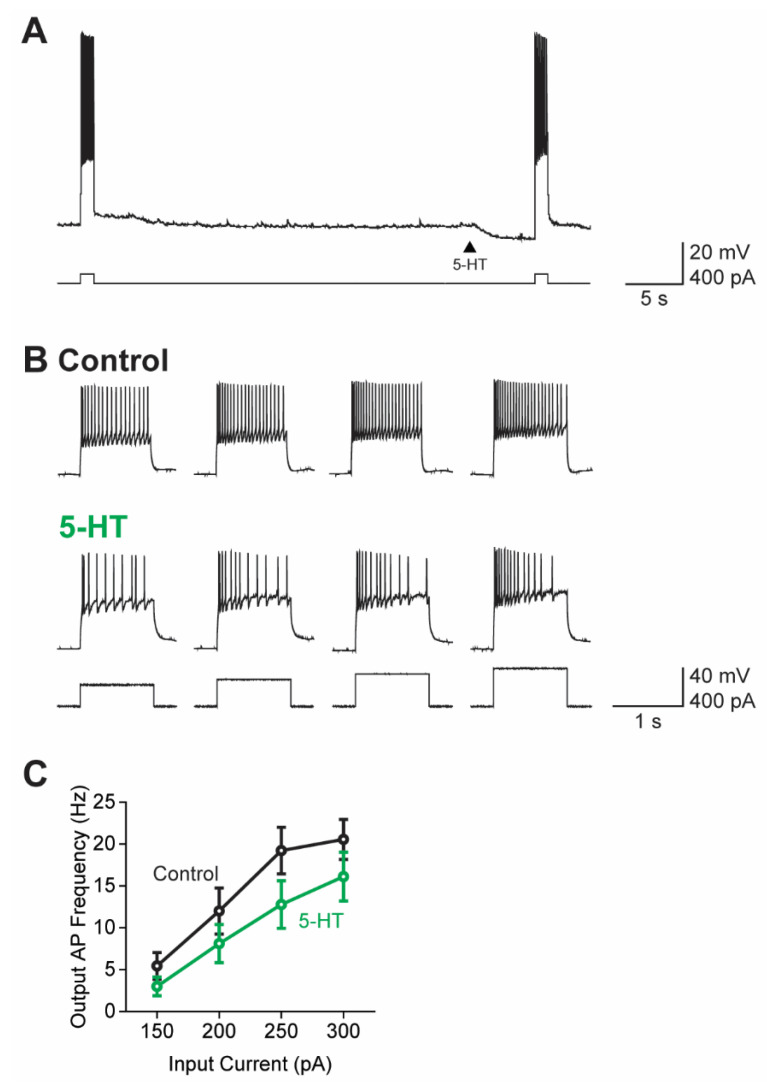
5-HT decreased the AP firing of claustral PNs. (**A**) Representative traces of AP firing (top) in a claustral PN in response to depolarizing current pulses (bottom). 5-HT (100 µM) was applied 5 s before the second depolarization (arrowhead) and hyperpolarized the membrane potential of the cell. (**B**) AP firing elicited by current pulses (bottom) before (Control; top) and after (center) 5-HT application. (**C**) Relationship between the magnitude of depolarizing current pulses and frequency of resulting APs in control conditions (black) and after application of 5-HT (green). Points indicate mean values and error bars show ±1 SEM.

As mentioned above, the 5-HT-mediated increase in K^+^ conductance should inhibit action potential (AP) firing, because the E_rev_ for the response (−95 mV; Figure 2D) was more negative than the AP threshold of all claustrum PNs (approximately −35 mV [39,40,71]). To determine the actions of 5-HT on AP firing, we examined claustrum PNs under current-clamp conditions; depolarizing current pulses were applied to evoke AP firing prior to and following the application of 5-HT (Figure 5A). This protocol allowed each neuron to serve as its own control and increased the robustness of our measurements. For this purpose, four rectangular current pulses (1 s duration) with amplitudes of 150, 200, 250, and 300 pA—well above the mean current threshold (CT) of PNs [40]—were delivered in the absence and presence of 5-HT (Figure 5B). In all experiments, 5-HT was applied 5 s prior to the depolarizations to ensure that current pulses aligned with the peak of the 5-HT-induced current.

Under these conditions, 5-HT hyperpolarized the resting membrane potential of PNs (Figure 5A) and inhibited AP firing (Figure 5B; *n* = 9). Although 5-HT reduced AP firing throughout each depolarizing current step, the initial burst of APs observed at the beginning of the depolarization [40,72] persisted in the presence of 5-HT (Figure 5B). To quantify the actions of 5-HT on AP firing, we determined the relationship between the amount of depolarizing current applied and the frequency of the resulting APs. Such input–output (I-O) curves showed that while larger depolarizations evoked more APs, 5-HT produced a consistent reduction in AP frequency at all stimulus levels (Figure 5C). In summary, we conclude that the increase in K^+^ conductance evoked by 5-HT does inhibit AP firing in claustral PNs.

**Figure 6:**
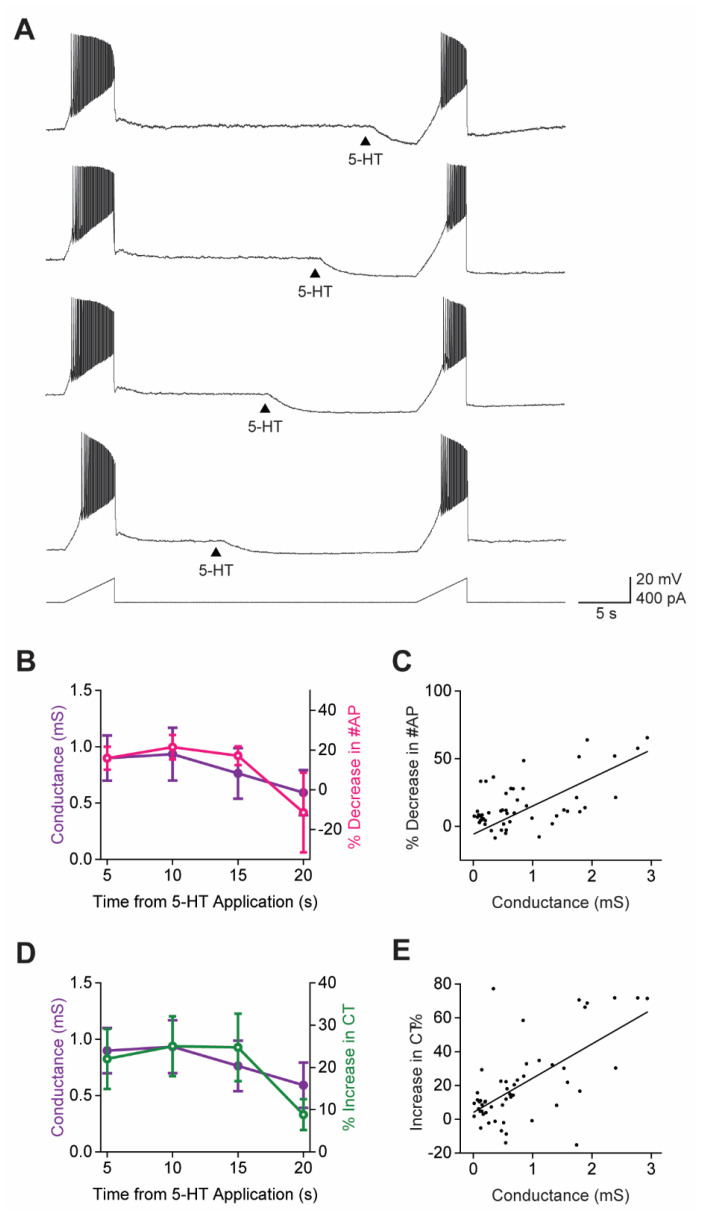
The temporal dynamics of AP firing reflects the time course of 5-HT-induced K^+^ conductance. (**A**) An example of claustral PN responses to pairs of depolarizing current ramps (bottom); 5-HT (100 µM) was applied at different times (arrowheads) prior to the second current ramp. (**B**) Relationship between the mean time course of 5-HT-induced K^+^ conductance (purple), measured in voltage-clamp conditions, and mean reduction in AP firing (pink) produced by application of 5-HT at variable times. Points indicate mean values, and error bars show ±1 SEM. (**C**) Correlation between the 5-HT-induced K^+^ conductance and reduction in AP firing measured in individual neurons. (**D**) Relationship between the mean time course of the 5-HT-induced K^+^ conductance (purple) and mean reduction in the AP current threshold (CT) produced by application of 5-HT at indicated times. Points represent mean values, and error bars show ±1 SEM. (**E**) Correlation between the 5-HT-induced K^+^ conductance and the reduction in CT (green) measured in individual neurons.

The characteristic time course of the 5-HT-induced K^+^ conductance in claustral PNs should affect the firing of APs in a time-dependent manner. To examine this possibility, we varied the time interval between application of 5-HT and depolarizing stimuli. Because PNs required long time intervals (approximately 1 min) to recover from each of the rectangular current pulses used in the experiments shown in Figure 5, we instead examined the timing of 5-HT effects by using ramps of depolarizing current to evoke APs (5 s duration, 400 pA peak amplitude; Figure 6A).

With this paradigm, we found that APs evoked by current ramps were maximally inhibited 5–10 s after 5-HT application (*n* = 13; Figure 6B, pink). The time course of the mean reduction in AP firing produced by 5-HT tracked the time course of the conductance changes induced by 5-HT (Figure 6B, purple). In individual experiments, the magnitude of the 5-HT-induced conductance change was also correlated with changes in the number of APs produced by 5-HT (Figure 6C; Pearson’s r = 0.39, *p* = 4.5 × 10^−3^). Similarly, 5-HT-induced changes in the CT tracked the time course of the 5-HT-induced conductance change (Figure 6D), and the magnitude of the 5-HT-induced CT changes in individual PNs was also correlated with the magnitude of the K^+^ conductance increase produced by 5-HT in each cell (Figure 6E; Pearson’s r = 0.64, *p* = 4 × 10^−7^). These results demonstrate that the 5-HT-induced changes in AP firing dynamics were time-locked to the 5-HT-induced K^+^ conductance, thereby providing additional evidence that this conductance was responsible for the observed inhibition of AP firing.

### 3.6. 5-HT Causes a Subtractive Reduction in PN Output

**Figure 7:**
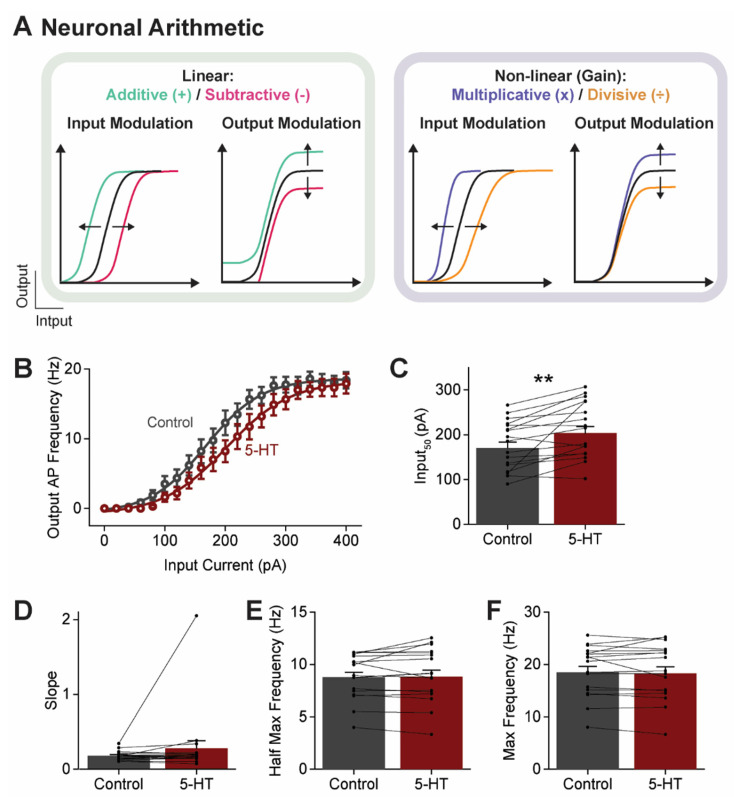
The subtractive action of 5-HT on neuronal arithmetic. (**A**) The input–output (I-O) relationship of a neuron could be modulated either linearly or non-linearly, changing processing of the input, the output or both. (**B**) I-O relationships of claustral PNs before (Control; black) or after (maroon) application of 5-HT (100 µM). Points indicate mean values and error bars show ±1 SEM. (**C**–**F**) I-O curve parameters obtained from Boltzmann function fits to the curves shown in (**B**): (**C**) Input_50_, (**D**) slope, (**E**) half-maximal AP frequency, and (**F**) maximum AP frequency. Points show individual measurements, bars indicate mean values, and error bars show SEM. Asterisks indicate a statistically significant difference (*p* = 0.0016); refer to Supplementary Table S1 for statistical analyses.

The modulation of neuronal activity can be characterized as an arithmetic operation [73,74]. The form of the I-O curve reveals whether such operations are linear or non-linear: a shift in the I-O curve along the x- or y-axes reflects linear additive or subtractive changes (Figure 7A, left), while changes in I-O curve slope indicate a non-linear multiplicative or divisive modulation of neuronal gain (Figure 7A, right). To assess the effect of serotonergic inhibition on claustral PN arithmetic, we examined how AP output changed in response to depolarizing current input. This experiment again relied on ramps of depolarizing current (5 s duration, 400 pA peak amplitude), as in Figure 6A, and 5-HT was applied 5 s prior to the current ramp, a time interval that yielded the maximal inhibition of AP firing (Figure 6B). APs evoked by the current ramps were binned into 20 epochs (250 ms each) to produce the I-O curves shown in Figure 7B, restricting our analysis to PNs that fired APs throughout the entire current ramp (*n* = 19).

Under control conditions, there was a sigmoidal relationship between current input and AP output (Figure 7B, black). This I-O curve was fitted with a Boltzmann function (Equation (3)), allowing quantification of control I-O parameters. Upon 5-HT application, the I-O curve shifted rightward, requiring a 32.3 ± 8.9 pA (23%) increase in current input to achieve the half-maximal AP output (Input_50_; Figure 7C). The magnitude and polarity of this shift was consistent with the 5-HT-induced outward current measured under voltage-clamp conditions at −70 mV (24.7 ± 4.6 pA) near the resting potential of PNs. The slope of the I-O curve was unchanged by 5-HT (Figure 7D), indicating no change in PN gain. Further, other parameters such as the half-maximal and maximal AP frequency were also unaffected (Figure 7E,F). These findings indicate that 5-HT induces a linear, subtractive decrease in claustral PN output, where a larger depolarizing current is required to reach the same AP output. Such an effect is consistent with the increased K^+^ conductance, and resulting hyperpolarization, elicited by 5-HT application.

### 3.7. 5-HTRs Are Distributed Throughout Claustral PN Compartments

In many brain regions, 5-HTRs are localized to different compartments of neurons, with such compartmentalization thought to influence neuronal output and contribute to the diversification of 5-HT responses [75,76]. To determine the location of 5-HTRs on claustral PNs, we focally uncaged 5-HT over PNs while measuring their electrical responses via patch-clamp recording. This approach has previously been employed to identify receptor location in neurons [77,78,79] and, in optimal conditions, can even map the distribution of receptors on individual dendritic spines [77,80]. Unlike anatomical techniques such as immunohistochemistry or the fluorescent tagging of receptors [21,25,59,60], this functional approach yields insights into both the subcellular location and biological actions of the receptors.

**Figure 8:**
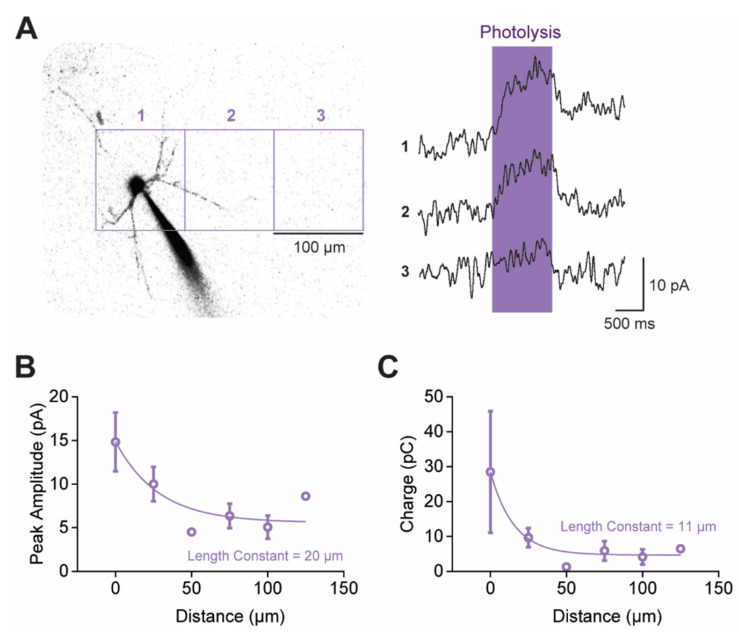
The spatial resolution of 5-HT uncaging. (**A**) Outward currents evoked by photolyzing caged 5-HT (10 µM) over a 100 µm-by-100 µm area at different locations, indicated by squares numbered 1–3. (**B**,**C**) Determination of the distance-dependence of uncaging, calculated from both the peak amplitude (**B**) and the charge (**C**) of the 5-HT-induced outward currents. Distance was calculated according to the distance to the nearest neighboring process of a neuron. Points indicate mean values and error bars show ±1 SEM. Lines represent fits of exponential functions to the data; the length constants of the exponential fits are also indicated.

In our experiments, individual claustral PNs were filled with a fluorescent dye to visualize their structure. 5-HT was then photoreleased by illuminating BHQ-O-5-HT (10 µM)—a light-sensitive caged compound that generates free 5-HT upon light absorption [44,45]—over visually identified compartments of the neuron. BHQ-O-5-HT was initially uncaged over a large area (100 µm by 100 µm) centered over PN somata (region 1 in Figure 8A). Light (405 nm) was applied for 1 s while holding the neuron at a membrane potential of −30 mV to increase the electrochemical driving force for K^+^ efflux and, thereby, improve the signal-to-noise ratio of the light-induced K^+^ current. To define the spatial resolution of uncaging, the light spot was systematically positioned away from the PN. The amplitude of responses to the uncaged 5-HT decreased as the light spot moved away from the PN cell body (regions 2 and 3 in Figure 8A). This effect was quantified by measuring the relationship between the amplitude of light-induced responses and distance from the PN, taking into account the position of dendrites within the uncaging area. Both the peak amplitude and charge of responses to the uncaged 5-HT decreased exponentially with distance away from the PN (*n* = 7; Figure 8B,C). The length constant of this exponential decay was 20 µm when measuring peak current amplitude and it was 11 µm for the less noisy measurements of response charge. Therefore, although the BHQ-O-5-HT was uncaged over a 100 µm-by-100 µm area, the effective range of free 5-HT was larger (approximately 122 µm by 122 µm, based on the more reliable charge measurements). For uncaging spots that were 10 µm by 10 µm in area, the length constant measured for response charge improved to 5 µm to yield an effective spatial range of approximately 20 µm by 20 µm (Supplementary Figure S3).

**Figure 9:**
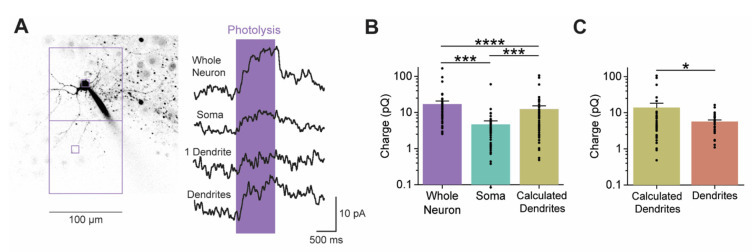
5-HT uncaging revealed the distribution of 5-HTRs in claustral PN neuronal compartments. (**A**) Outward currents evoked by photolyzing caged 5-HT (10 µM) over nearly the whole neuron (upper large square in left image), soma (upper small square in left image), 1 dendrite (lower small square in left image), or many dendrites (lower large square in left image). (**B**) The charge of outward currents induced by uncaging 5-HT at the whole neuron, soma, and calculated dendrite component (whole neuron–soma). (**C**) The charge of the 5-HT-induced outward currents for the calculated dendrite component and actual responses measured after uncaging 5-HT over a large area that included many dendrites. Points in (**B**,**C**) show individual measurements, bars indicate mean values, and error bars show SEM. Asterisks indicate statistically significant differences; refer to Supplementary Table S1 for statistical analyses. * *p* ≤ 0.05, *** *p* ≤ 0.001, **** *p* ≤ 0.0001.

For 100 µm-by-100 µm light spots centered over PN cell bodies (Figure 9A, left), the uncaged 5-HT reached a significant fraction of the spatial extent of a typical claustral PN [3]. We therefore refer to responses to uncaging BHQ-O-5-HT over such areas as “whole neuron” responses (Figure 9A, right). These responses were observed in 92.6% (50/54) of claustral PNs examined. They had a peak amplitude of 13.9 ± 1.0 pA (mean ± 1 SEM), had a mean charge of 17.0 ± 3.8 pC, and decayed exponentially, with a time constant of 0.7 ± 0.2 s. These parameters are different from those of the responses to pressure-applied 5-HT described above. Probably 5-HT uncaging yielded a higher response rate because the depolarized holding potential used enhanced the detection of small K^+^ conductance responses. Changes in other response properties likely resulted from the more restricted volume of 5-HT delivery produced by BHQ-O-5-HT uncaging.

To improve the spatial resolution for localizing 5-HTRs, we next used the smaller uncaging spot (10 µm by 10 µm). When these smaller light spots were used to uncage BHQ-O-5-HT over the soma of claustral PNs (red square in Figure 9A, left), very small outward currents were elicited (*n* = 50; “soma” in Figure 9A, right). These currents were typically only a few pA in peak amplitude, had a charge of 4.5 ± 1.2 pC, and had a decay time constant of 0.6 ± 0.1 s. These results indicate that 5-HTRs are present on the soma of PNs. However, when we used similar light spots to uncage BHQ-O-5-HT over individual dendrites (small purple square in Figure 9A, left), no 5-HT-induced outward currents were detectable (“1 dendrite” in Figure 9A, right). Because the responses to uncaging BHQ-O-5-HT over most of the neuron were several-fold larger than those produced by uncaging only over somata, we conclude that dendrites must also have 5-HTRs. However, it is likely that the poor signal-to-noise ratio of the responses to uncaged BHQ-O-5-HT prevented our detection of the tiny currents likely to be produced by uncaging BHQ-O-5-HT over thin, individual dendrites.

To assess 5-HTRs on the dendrites of claustral PNs, we compared “whole neuron” responses to those measured when BHQ-O-5-HT was uncaged only over the soma. The charge associated with responses to somatic uncaging was 31% of that of the “whole neuron” responses, indicating that at least 69% of the response to uncaged 5-HT originated from a non-somatic compartment (Figure 9B). The difference between the “whole neuron” and “soma” responses should approximately reflect the dendritic contribution to the response to uncaged 5-HT; we therefore refer to this component as the “calculated dendrite” response in Figure 9B (*n* = 50). Consistent with this assumption, uncaging 5-HT over areas that included dendrites but excluded somata (large purple square in Figure 9A, left) evoked measurable responses (“dendrites” in Figure 9A, right). The mean charge of these responses was 5.7 ± 0.6 pC, which was roughly one-third of the magnitude of the “calculated dendrite” responses (Figure 9C). This difference probably arose from the 5-HT being uncaged over only a fraction of the PN dendritic arbor during the “dendrite” responses. In summary, 5-HTRs are present on PN dendrites. Indeed, our measurements indicate that most 5-HTRs are located in the dendritic compartment; this likely reflects the nearly 10-times larger surface area of PN dendrites compared to their somata [81].

**Figure 10:**
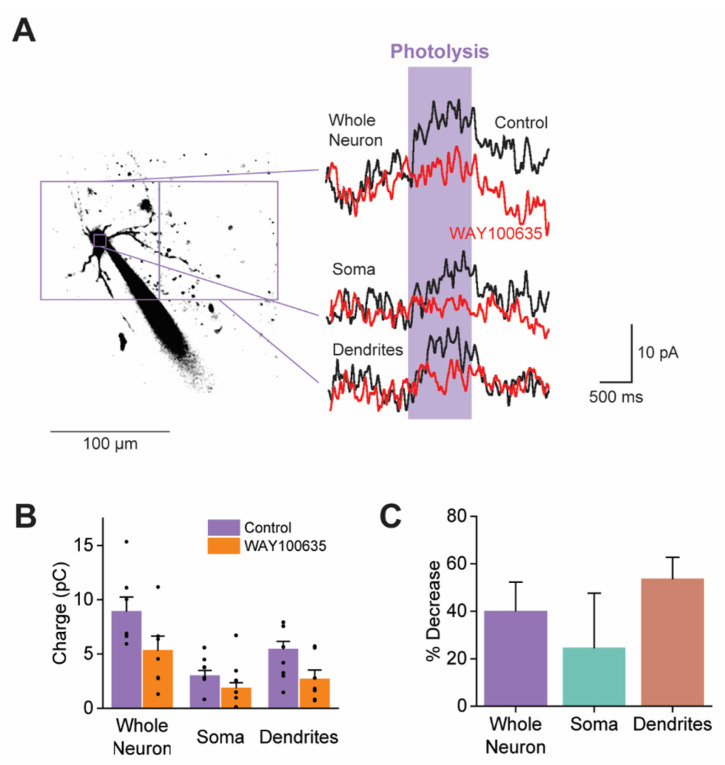
The localization of 5-HTR-1A on claustral PNs. (**A**) Outward currents evoked by photolyzing caged 5-HT (10 µM) over the whole neuron (large left square), soma (small square), and dendrites (large right square). Black traces represent responses measured in normal ACSF, while red traces represent responses measured in the presence of WAY100635 (1 µM). (**B**) The total charge of responses to uncaging 5-HT in the indicated neuronal compartments, measured in control conditions and in the presence of WAY1000635. Points show individual measurements, bars indicate mean values, and error bars show SEM. (**C**) The percentage reduction in responses to uncaging 5-HT produced by WAY1000635. Bars indicate mean values, and error bars show SEM. Refer to Supplementary Table S1 for statistical analyses.

To identify the 5-HTR subtypes mediating these responses, we examined the effect of the 5-HTR-1A antagonist WAY100635 (1 µM) on the somatic and dendritic responses to uncaged 5-HT. 5-HTR-1A is the primary contributor to the 5-HT-induced K^+^ conductance (Figure 4). Because PN recordings could only be maintained for a limited time, while antagonist application required several minutes, uncaging of BHQ-O-5-HT was performed at a time when the concentration of WAY100635 at the PNs may not yet have reached 1 µM. Nonetheless, the WAY100635 reduced responses to uncaged BHQ-O-5-HT (*n* = 7; Figure 10A). This effect was observed across all PN compartments: in all cases, the 5-HT-induced outward current was smaller in the presence of WAY100635 compared to control conditions (Figure 10B). Thus, 5-HTR-1A is present on both PN somata and dendrites. While the dendritic responses were apparently blocked more strongly than the somatic responses (Figure 10C), this difference was not statistically significant (Supplementary Table S1). Responses in none of these compartments were completely blocked by WAY100635. This could be due to the presence of other 5-HTRs, presumably 2A and 2C subtypes, on the somata and dendrites of claustral PNs and/or due to insufficient time allowed for the WAY100635 to act fully.

## 4. Discussion

We have found that the output neurons of the claustrum are inhibited by 5-HT and have identified the ionic mechanism and receptors involved in this inhibition. Whole-cell patch-clamp recordings from over 180 claustral PNs revealed that serotonergic inhibition is caused by a K^+^ conductance increase that reduces neuronal excitability through hyperpolarization, making it more difficult for neurons to fire action potentials. Comparison of the effects of 5-HTR antagonists indicated that the inhibitory action of 5-HT is primarily mediated by 5-HTR-1A, with some contributions from 5-HTR-2A and 5-HTR-2C. Using caged 5-HT, we also identified the presence of 5-HTRs on both the somata and dendrites of claustral PNs.

### 4.1. Ionic Mechanism of 5-HT Inhibition of Claustrum PNs

Most claustral PNs generated a long-lasting K^+^ current in response to 5-HT. The voltage dependence of this 5-HT response indicates that the current rectified in the inward direction (Figure 2D) and that its rectification properties depended upon the K^+^ electrochemical gradient (Figure 2E). These properties definitively establish that this response was mediated by inward rectifier K^+^ channels. However, seven different K^+^ channel gene families generate inwardly rectifying K^+^ currents [82,83,84], and it is not yet clear which type of inward rectifier K^+^ channel is responsible. We have searched for inward rectifier K^+^ channel genes (Kcnj) in the claustral single-cell transcriptomic data of Erwin et al. [85]. Our analysis indicates that in the claustrum core, where our measurements of 5-HT responses were made, at least eight inward rectifier channel genes are expressed: Kcnj2, 3, 4, 5, 6, 9, 11, 14. Among these are G-protein-coupled inward rectifier K^+^ channels (GIRKs), which are known to mediate inhibitory 5-HT responses in other neurons [55,83,86,87]. All four GIRK genes are expressed in the claustrum core: GIRK1 (Kcnj3), GIRK2 (Kcnj6), GIRK3 (Kcnj9), and GIRK4 (Kcnj5). It is notable that while GIRK4 is rarely expressed in the brain, it is abundant in the claustrum. Given this diversity of candidate genes, and the ability of their gene products to heterotetramerize [88,89], it is clear that a substantial effort will be required to identify the specific K^+^ channel genes that underlie the 5-HT response of claustral PNs.

To understand how the activation of inward rectifier K^+^ channels by 5-HT affects information coding in claustral PNs, we determined the effects of 5-HT on AP firing and the I-O functions of PNs. Our experiments showed that 5-HT inhibits PN output by increasing the threshold current required to trigger an AP, thereby reducing AP firing rate. These effects are due to 5-HT hyperpolarizing the resting membrane potential and decreasing the input resistance [90,91,92]. Collectively, these effects were reflected in a rightward shift in the I-O curve, causing a subtractive effect on PN information coding. The continued presence of burst spikes [40,72] during 5-HT inhibition suggests that 5-HT does not modulate the ionic conductances responsible for burst spiking [93] and, instead, works entirely via inwardly rectifying K^+^ channels.

The 5-HT-induced K^+^ current was relatively stereotyped across all claustrum PNs (Figure 3C). The main difference between the responses of the PN subtypes was their probability of responding to 5-HT (Figure 3B), which presumably reflects differences in their 5-HT receptor expression. Such genetic and epigenetic control of claustral 5-HTR expression in PN subtypes will be an important topic for future research. Whatever the source of this response heterogeneity, it has important implications for serotonergic modulation of the claustrum. In particular, we found that the PN1 subtype of projection neuron is less frequently inhibited by 5-HT. Because these are the only claustral PNs known to target subcortical structures [40], this means that 5-HT action within the claustrum is likely to selectively inhibit claustrum output to cortical structures while sparing output to subcortical structures. A similar “toggle” mechanism has also been proposed for GABA co-released from cholinergic fibers innervating the claustrum [71] and suggests that biasing claustrum output toward or away from the cortex is a general scheme for neuromodulator action.

### 4.2. Receptors Mediating 5-HT Inhibition

Type 1A, 2A, and 2C 5-HTRs are metabotropic receptors that are most likely involved in the 5-HT response of claustral PNs, based on their expression levels in the claustrum and their established roles in 5-HT signaling elsewhere in the brain. Among these receptor types, our pharmacological analysis indicates that all three contribute to the 5-HT response of claustral PNs, with 5-HTR-1A mediating most of this response. Application of specific 5-HT agonists revealed that serotonergic inhibition in the lizard claustrum is mediated by 5-HTR-1D [18]; this indicates an evolutionarily conserved function for claustral 5-HTR-1 in both mammals and reptiles. Our findings align with previous reports of inhibitory 5-HT responses in other types of mammalian neurons: 5-HTR-1A inhibits various neurons by activating inward rectifier K^+^ channels, in particular GIRK channels [57,58,88,94]. The role of 5-HTR-1A in the observed subtractive effect of 5-HT on claustral PNs is consistent with findings that 5-HTR-1A hyperpolarizes and suppresses spontaneous activity in mouse visual cortical neurons [93,95].

While 5-HTR-2A and 5-HTR-2C are typically associated with excitatory responses in many cortical regions [96,97], we found evidence that both receptors contribute to inhibitory 5-HT responses in claustral PNs. This is consistent with growing indications of 5-HTR-2A-mediated inhibitory responses in other brain areas, such as the anterior piriform cortex [98] and prefrontal cortex [99]. 5-HTR-2A and 5-HTR-2C may also contribute to the subtractive effect of 5-HT in claustral PNs, though apparently to a lesser extent.

Although the slow outward current produced by 5-HT was rather consistent across claustrum PNs (Figure 3C), we found that the contribution of each 5-HTR type to this response was surprisingly variable across PNs (Figure 4B). Our data indicate that this heterogeneity probably cannot be explained simply by the diversity of PN subtypes (Supplementary Figure S2). Comparing our results with single-cell transcriptome data from mouse claustral PNs [85], we found that 5-HTR-1A, 2A, and 2C are heterogeneously expressed in individual claustrum neurons; this may account for the variability in responses to 5-HTR antagonists that we observed. Recent evidence suggests the presence of functional intracellular 5-HTR-2A in neurons [100], which could explain why the high apparent expression of 5-HTR-2A in the claustrum does not lead to a 5-HTR-2A-dominant response of PNs to extracellular 5-HT. Another potential source of variability is mRNA editing of 5-HTR-2C, which can produce multiple isoforms with potentially different physiological effects [19,93,101,102]. Still other sources of variability may come from downstream signaling cascades—which vary for different types of 5-HTRs [93,103]—and/or the sensitivity of different types of inward rectifier K^+^ channels to different G-protein subunits and second messengers [83]. Finally, the heterodimerization of 5-HTRs with other 5-HT or non-5-HT receptors may activate different G-proteins, leading to diverse downstream effects [104].

### 4.3. Location of 5-HT Receptors on Claustrum PNs

Until now, there have been no analyses of 5-HTR-1A localization across different compartments of claustrum neurons. To link the location of 5-HTRs with their functional output, we combined electrophysiology with the focal uncaging of 5-HT on individual PNs. This is the first time focal uncaging has been applied to the analysis of 5-HTR localization for any neuron. We found that this uncaging approach to localizing 5-HTRs suffers from a low signal-to-noise ratio. Uncaging BHQ-O-5-HT for relatively long times, and over relatively large volumes, improved the signal-to-noise ratio but reduced the effective spatial resolution. Nonetheless, the resolution was adequate to reveal the general location of 5-HTRs on claustrum PNs. Specifically, we found that 5-HTRs are present on both the cell bodies and dendrites of these neurons. The larger outward current responses observed when uncaging BHQ-O-5-HT over groups of dendrites, compared to the smaller current at the soma, likely reflected the greater surface area of the dendrites [81]. Further, we found that at least part of these responses on both PN compartments were mediated by 5-HTR-1A. This differs from findings in neurons in the prefrontal cortex, where 5-HTR-1A are compartmentalized to either somata or dendrites [75,76].

Although the exact site of 5-HT release onto claustral PNs is unknown, 5-HT likely broadly affects these neurons through volume transmission, diffusing widely in the extracellular space [105]. This suggests that in vivo, synaptic release of 5-HT activates 5-HTR-1A on both dendrites and somata, causing widespread hyperpolarization. As a result, stronger excitatory synaptic input would be required on the dendrites to generate the same output at the soma, resembling the effects on neuronal arithmetic observed when interneurons inhibit cortical PNs [106,107,108]. Thus, the broad distribution of 5-HTRs in claustral PNs likely explains the observed subtractive effects of 5-HT on claustral neuronal arithmetic.

### 4.4. Implications for Higher Brain Function

The actions of neuromodulators, such as 5-HT, in the claustrum are likely to play many important roles in brain function [12]. Thus, our results will lay the foundation for a better understanding of claustrum functions in the future. Here, we will briefly provide two examples, already raised in the Introduction, that illustrate this point. First, the claustrum is known to be involved in slow-wave sleep [17,18]. Because 5-HT levels are low during this phase of sleep, it has been proposed that the absence of 5-HT inhibition enables sharp-wave ripples to be generated in the claustrum, with slow-wave sleep then terminated when elevated 5-HT levels inhibit the claustrum [18]. Our results provide a mechanistic understanding of this inhibitory action of 5-HT and, thereby, advance our understanding of the roles of both 5-HT and the claustrum in regulation of sleep. Second, many psychedelic compounds are thought to act via 5-HTR-2A [34,35]. Given that the claustrum has a high density of these receptors [12], it has been proposed that the claustrum may be involved in the hallucinations produced by such compounds [36,37,109]. Our results establish that there are functional 5-HTR-2A in the claustrum and that these receptors principally act by inhibiting communication between the claustrum and the cortex, due to 5-HT-2A hyperpolarizing cortically projecting PNs.

## 5. Conclusions

We have found that 5-HT inhibits claustral PNs and does so by activating inward rectifier K^+^ channels. This inhibitory action of 5-HT reduces the ability of claustral PNs to respond to excitatory stimuli, specifically producing a subtractive effect that increases the amount of depolarizing current required to evoke APs without affecting the maximum number of APs that can be produced by a PN. We have also determined that type 1A, 2A, and 2C 5-HTRs mediate this inhibition. By establishing a method to visualize 5-HTR localization, using caged 5-HT and whole-cell patch-clamp recording, we found that 5-HTR-1A are distributed throughout claustral PNs. Further research on serotonergic inhibition in the claustrum, particularly regarding 5-HT release and its effects in vivo, will deepen our understanding of the claustrum.

While this paper was in preparation, two other groups reported results consistent with ours. An abstract has indicated that 5-HT inhibits excitatory synaptic transmission between the cortex and claustrum [110], supporting our findings of widespread 5-HT inhibition of claustral PNs. A very recently published paper [111] reports that 5-HT hyperpolarizes claustrum neurons and inhibits their AP firing, effects that are mediated by multiple 5-HTR types. We have observed very similar effects on claustrum PNs and have elucidated the ionic mechanism underlying these effects, as well as identifying a subtractive action of 5-HT on PN information processing and establishing the localization of 5-HTRs on PN somata and dendrites.

## Data Availability

Upon the acceptance of this paper, data will be deposited in a publicly accessible database.

## Supplementary Materials

The following supporting information can be downloaded at https://www.mdpi.com/article/10.3390/cells13231980/s1: Figure S1: Lack of polysynaptic component of 5-HT responses; Figure S2: Effects of 5-HTR antagonists on PN subtypes; Figure S3: Spatial resolution of 5-HT uncaging for a 10 µm-by-10 µm area of photolysis; Supplementary Table S1: Statistical analysis for data contained in this paper.
