# Supplementary material for: Serotonin Inhibition of Claustrum Projection Neurons: Ionic Mechanism, Receptor Subtypes and Consequences for Claustrum Computation
Source: Cells. 2024 Nov 29;13(23):1980. doi: 10.3390/cells13231980 (PMC11640313; doi:10.3390/cells13231980)
Supplement: Supplementary file 1 [file cells-13-01980-s001.zip › cells-3316897-supplementary.pdf]

Supplementary Table S1. Statistical analysis for data contained in this paper.

Figure	Group(s) and Sample Size	Test/Fit	Result
Suppl. Figure S1	Control vs. KA + GBZ for charge (*n* = 13)	Paired *t*-test	t(12) = 1.47, $p = 0.17$
Figure 3B	Overall response rate (*n* = 182)	Chi square	$X^2_{(1, 182)} = 25.1$, $p = 0.00005$
	PN1 (*n* = 19) vs. average response rate (*n* = 182)		$X^2_{(1, 201)} = 7.12$, $p = 0.008$
	PN2 (*n* = 42) vs. average response rate (*n* = 182)		$X^2_{(1, 272)} = 0.001$, $p = 0.974$
Figure 3C	PN subtype effect on peak amplitude (*n* = 182)	Welch's ANOVA	$F_{(4, 123)} = 3.53$, $p = 0.023$
	Pairwise comparison	Tukey's test	$p > 0.05$
Related to Figure 3	PN subtype effect on charge (*n* = 182)	Welch's ANOVA	$F_{(4, 123)} = 7.77$, $p = 0.0010$
	Pairwise comparison	Tukey's test	$p > 0.05$
Related to Figure 3	PN subtype effect on exponential decay (*n* = 182)	Welch's ANOVA	$F_{(4, 122)} = 0.35$, $p = 0.84$
Related to Figure 3	PN subtype effect on peak time (*n* = 182)	Welch's ANOVA	$F_{(4, 123)} = 0.26$, $p = 0.902$
Related to Figure 3	PN subtype effect on conductance (*n* = 182)	Welch's ANOVA	$F_{(4, 123)} = 3.53$, $p = 0.023$
	Pairwise comparison	Tukey's test	$p > 0.05$
Figure 4B	Drug effect on % decrease in charge (*n* = 41)	Welch's ANOVA	$F_{(2, 38)} = 15.818$, $p = 0.00001$
	Pairwise comparison	Tukey's test	1A vs. 2A: $p < 0.05$<br>1A vs. 2C: $p < 0.0001$<br>2A vs. 2C: $p = 0.21$
Figure 7C	Control vs. 5-HT for $input_{50}$ (*n* = 19)	Paired *t*-test	$t_{(18)} = -3.70$, $p = 0.0016$
Figure 7D	Control vs. 5-HT for slope (*n* = 19)	Paired *t*-test	$t_{(18)} = -1.12$, $p = 0.28$
Figure 7E	Control vs. 5-HT for $output_{50}$ (*n* = 19)	Paired *t*-test	$t_{(18)} = -0.24$, $p = 0.82$
Figure 7F	Control vs. 5-HT for $output_{Max}$ (*n* = 19)	Paired *t*-test	$t_{(18)} = 0.59$, $p = 0.56$
Figure 9B	Location effect on Charge (*n* = 50)	One-way repeated ANOVA	$F_{(2, 147)} = 4.89$, $p = 0.008$
	Whole neuron vs. soma for charge (*n* = 50)	Paired *t*-test	$t_{(49)} = 4.31$, $p = 0.00008$
	Whole neuron vs. calculated dendrites for charge (*n* = 50)	Paired *t*-test	$t_{(49)} = 3.91$, $p = 0.0002$
	Soma vs. calculated dendrites for charge (*n* = 50)	Paired *t*-test	$t_{(49)} = 3.48$, $p = 0.001$
Figure 9C	calculated dendrites vs. dendrites for charge (*n* = 31)	Paired *t*-test	$t_{(30)} = 2.32$, $p = 0.03$
Figure 10B	Main effect of Drug for 1 s Integral (*n* = 7)	Two-way repeated ANOVA	$F_{(1, 38)} = 11.64$, $p = 0.002$
	Main effect of Location Current for 1 s Integral (*n* = 7)		$F_{(2, 38)} = 14.10$, $p = 0.00003$

	Interaction between Drug and Location for 1 s Integral ($n = 7$)		$F_{(1, 38)} = 13.28$, $p =$ 0.000004
Figure 10C	Location effect on Charge ($n = 7$)	One-way repeated ANOVA	$F_{(2, 18)} = 0.93$, $p = 0.41$

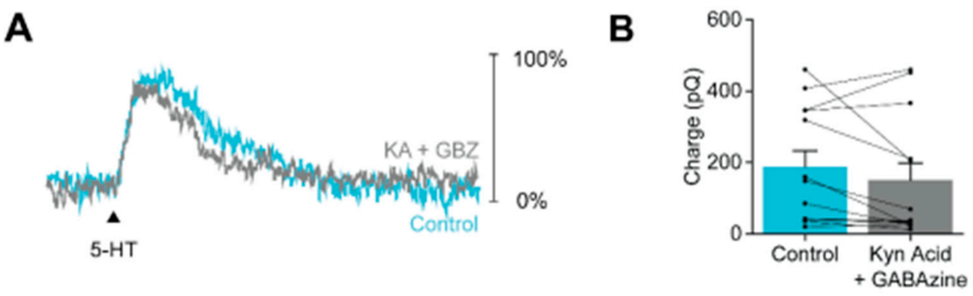

Supplementary Figure S1. Lack of polysynaptic component of 5-HT responses. (A) Representative traces of 5-HT responses of the same PN with and without antagonists kynurenic acid (KA) and GABAzine (GBZ). (B) Mean charge of 5-HT responses was not significantly different in the absence and presence of antagonists (refer to Table 1 for statistical analysis). Bars indicate mean values and error bars show SEM.

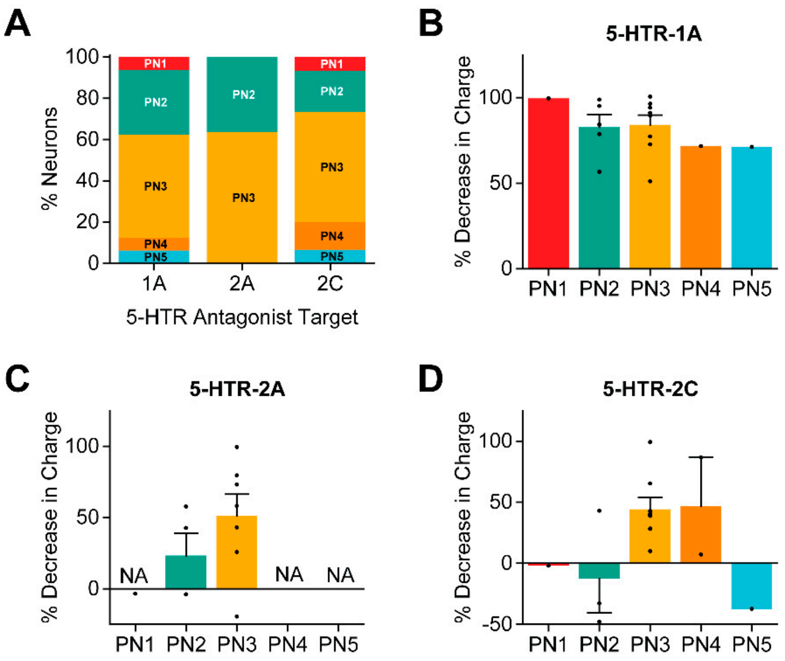

Supplementary Figure S2. Effects of 5-HTR antagonists on PN subtypes. (A) Proportion of various claustral PN subtypes (PN1 to PN5) in the dataset. (B-D) Decrease in charge sorted by claustral PN subtypes post-drug application: (B) 5-HTR-1A antagonist WAY100635 (1 μM), (C) 5-HTR2A antagonist MDL11939 (5 μM), and (D) 5-HTR-2C antagonist RS102221 (5 μM).

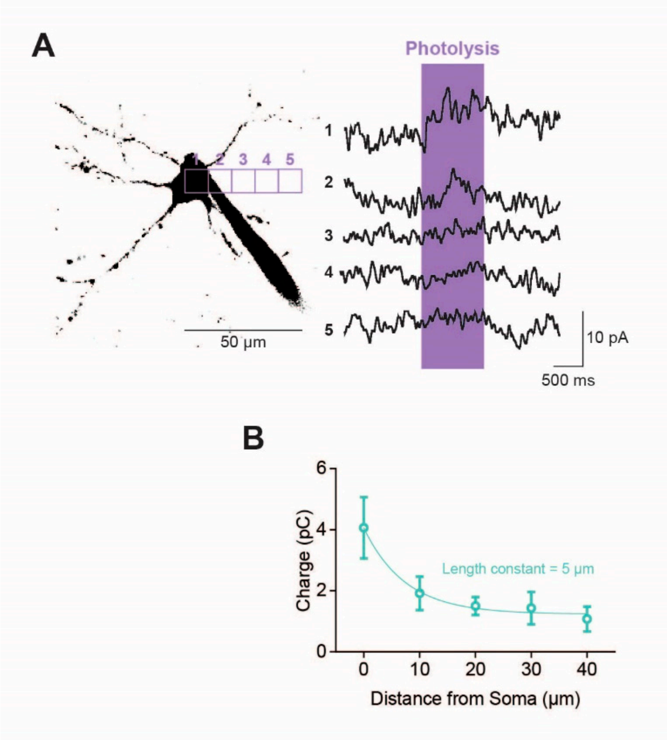

Supplementary Figure S3. Spatial resolution of 5-HT uncaging for a 10 μm by 10 μm area of photolysis. (A) outward currents caused by photolyzing caged 5-HT (10 μM) over a 10 μm by 10 μm square area at different distances away from the soma. (B) Charge of the 5-HT-induced outward currents plotted against distance from soma. Points indicate mean values and error bars show ±1 SEM.
